# Unrevealing tunable resonant excitons and correlated plasmons and their coupling in new amorphous carbon-*like* for highly efficient photovoltaic devices

**DOI:** 10.1038/s41598-023-31552-5

**Published:** 2023-05-04

**Authors:** D. Darminto, Retno Asih, Budhi Priyanto, Malik A. Baqiya, Irma S. Ardiani, Khoirotun Nadiyah, Anna Z. Laila, Soni Prayogi, Sarayut Tunmee, Hideki Nakajima, Angga D. Fauzi, Muhammad A. Naradipa, Caozheng Diao, Andrivo Rusydi

**Affiliations:** 1 Department of Physics, Institut Teknologi Sepuluh Nopember, Surabaya, 60111 Indonesia; 2 Department of Electrical Engineering, Muhammadiyah University, Malang, 65145 Indonesia; 3 Synchrotron Light Research Institute, 111 University Avenue, Muang District, Nakhon Ratchasima, 30000 Thailand; 4 Advanced Research initiative for Correlated-Electron Systems (ARiCES), Department of Physics, National University of Singapore, Singapore, 117542 Singapore; 5 Singapore Synchrotron Light Source, National University of Singapore, 5 Research Link, Singapore, 117603 Singapore

**Keywords:** Materials science, Physics

## Abstract

An understanding on roles of excitons and plasmons is important in excitonic solar cells and photovoltaic (PV) technologies. Here, we produce new amorphous carbon (*a*-C) *like* films on Indium Tin Oxide (ITO) generating PV cells with efficiency three order of magnitude higher than the existing biomass-derived *a*-C. The amorphous carbon films are prepared from the bioproduct of palmyra sap with a simple, environmentally friendly, and highly reproducible method. Using spectroscopic ellipsometry, we measure simultaneously complex dielectric function, loss function as well as reflectivity and reveal coexistence of many-body resonant excitons and correlated-plasmons occurring due to strong electronic correlations. X-ray absorption and photoemission spectroscopies show the nature of electron and hole in defining the energy of the excitons and plasmons as a function of N or B doping. Our result shows new *a*-C *like* films and the importance of the coupling of resonant excitons and correlated plasmons in determining efficiency of photovoltaic devices.

## Introduction

Photovoltaic (PV) technologies offer practical and efficient ways for harnessing sunlight, the most abundant, clean, and safe energy source, into electricity^1,2^. This is a promising technology to generate a huge scale of electrical power^3,4^. Global cumulative photovoltaic capacity has grown continuously since 2000 and amounted to 633.7 GW in 2019, showing that global markets have been shifted toward renewable energy^5^. Such demands and trends have driven research in PV technology progressively, including materials used, designs of PV panels, as well as the efficiency. However, the efficiency of PV cells (PVCs) faces limitations^6,7^, particularly in producing power that competitive with that of fuel-cells technology. Improvements of performance and efficiency are necessary, crucially on the development of functional materials, as alternatives of silicon (Si). Therefore, understanding the mechanism to generate power conversion efficiency and searching for new materials for PV devices are critical.

Carbon (C) is expected to have resemblant properties as Si and would be exceptionally stable^8,9^. Carbon exists in a variety of stable allotropes with diverse properties, ranging from insulating found in diamond, to semiconducting in fullerenes and metallic in graphene^8–11^. Among carbon polymorphs, amorphous carbon (*a*-C) has attracted a great interest due to the feasibility of controlling its conducting type, which makes it possible as an alternative material in optoelectronic devices^8,9,12^. Properties of PV based on *a*-C can be altered over a wide range^8^. Its band gaps can be commanded in a relatively broad range of 0.2–3.0 eV^8^ by controlling the electronic structure, including the ratio of *sp*^*2*^/*sp*^3^ hybridizations, and dopants amounts^13–15^. Several studies on *a*-C/Si junction showed a possible realization of *a*-C based solar cells^15–18^. Moreover, *a*-C films offer an additional feature as a protective antireflecting coating for Si solar cells, thus can enhance the cell efficiency^19^. However, our understanding on the electronic and optical structures of the *a*-C is very limited.

In thin-film PVCs technologies, small absorbance of near-bandgap light is the main limitation. A recent method has then been proposed to increase light trapping by using metallic nanostructures to support surface plasmons^20^. Plasmon, a quantum of plasma oscillation, is expected to improve the capability to absorb incoming light and produce electric charges by concentrating the electromagnetic field into the active region within specific spectral regions^21^. These regions can be tuned by the size, shape, and distribution of plasmonic particles and their surrounding medium^20,21^. This implies that plasmonic emerges as a new area and plays a key role for PV devices. On the other hand, the presence of excitons, a bound electron–hole pair, is a fundamental aspect, particularly for excitonic PV to generate charge carriers. Recently, a type of new exciton so-called resonant exciton^10,22,23^, and a type of new plasmon so-called correlated-plasmon^24^, have been observed in correlated electron systems. The existence of these two quasiparticles, excitons and plasmons, with a strong coupling in a PVC could induce Fano resonance^25,26^, which then provides an efficient channel of coherent energy transfer from metallic plasmons to molecular excitons^25^. This approach may offer a new strategy to design PV nanodevices. However, so far, there were no report on the resonant excitons and correlated-plasmons and little systematic thought has been given to the question of how such plasmons and excitons might be used beneficially in PV technologies. Here, we report that many-body resonant excitons and correlated-plasmons simultaneously present in a new *a*-C *like* film on ITO, and their coupling is essential to generate PV characteristics.

## Methods

### Film preparations

The first step to making *a*-C films is preparing carbon powders from palmyra sap. The palmyra sap is caramelized by heating at 100 °C while stirring it at 200 revs/min. The caramel is then calcined at 250 °C for 2.5 h inside a furnace. The obtained carbon powder is ultrasonically cleaned in distilled water for 30 min, and the solution is filtered to get the carbon sediment. This process is repeated three times to remove KCl salt altogether. After drying, grinding, and sieving the sediment, fine powders of *a*-C is achieved. To prepare *a*-C:B, H_3_BO_3_ (Merck, 99.5%) is dissolved in distilled water at 170 °C, and the solution is mixed with fined carbon powder with the mol ratio of B:C is 1:5. The mixing process is performed at 300 °C with the stirring speed of 200 revs/min until the mixed solution dries up. The same method is applied to prepare *a*-C:N, except for that, in this case, carbon powder is dissolved into NH_4_OH 1 M (Merck, 25%) with the mol ratio of N:C is 1:5.

The next step is to prepare carbon solutions used for fabricating the films. Fine powders of *a*-C, *a*-C:B, and *a*-C:N are separately dissolved in the mixed solution of Dimethyl Sulfoxide (DMSO, Merck, 99.9%) and distilled water with the ratio of 1:10. The solution is then mixed in an ultrasonic cleaner for two hours, and the result is centrifuged at 3500 revs/min for 45 min to achieve homogeneous carbon solutions. Carbon films of *a*-C, *a*-C:N, and *a*-C:B are prepared by depositing carbon solutions on the ITO substrate by a nano-spray method. This method offers more homogenous deposited films compared to that of a spin-coating technique^27^. The size of ITO is 2 × 1 cm^2^, and it has been ultrasonically cleaned in alcohol for one hour before the deposition process. The distance between the spray and ITO is fixed to be 5 cm, and the deposition time is set for 10 s.

### Characterizations

The phase and crystal structure of the obtained carbon films are examined by a Phillips X’Pert Multipurpose diffractometer with Cu *K*α radiation (*λ* = 1.5406 Å) at room temperature. Scanning electron microscopy with energy dispersive x-ray (SEM–EDX, EVO® MA 10) is employed to investigate the film's surface structure, elemental composition, and thickness. The structure of amorphous carbon is also investigated using Raman spectroscopy (SENTERRA) at wavenumber of 50–3500 cm^−1^. X-ray photoemission spectroscopy (XPS) measurements are performed at beamline BL3.2a in Synchrotron Light Research Institute (SLRI), Thailand. The XPS spectra are collected at room temperature and calibrated against the C1s signal from a conductive carbon tape. The soft x-ray absorption spectra of the films are collected at the SUV beamline of the SSLS synchrotron facility, National University of Singapore. Details of the instrument and measurement geometry can be found elsewhere^28^.

Parameters Ψ and Δ of spectroscopy ellipsometry are gathered at 50, 60, 70, and 80° angles of incidence, with a photon energy range between 0.62 and 5.62 eV using a Woollam V-vase ellipsometer. Details of the instrument are described elsewhere^29^. The obtained spectra are fitted with Woollam Complete Ease software^30^. The dielectric constant is extracted using least-squares regression analysis and an unweighted root-mean-square error function, where a combination of PSemi-Tri oscillator functions evaluates its parametrization.

## Results and discussions

**Figure 1:**
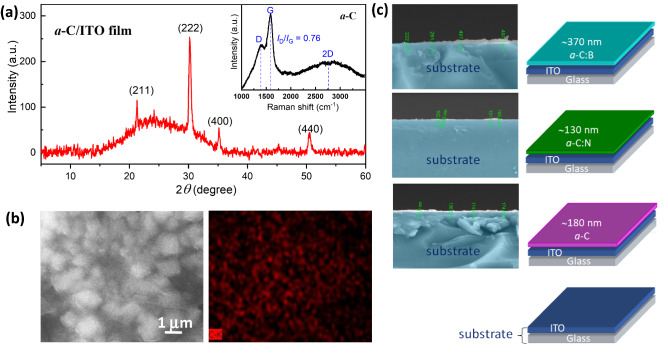
(**a**) The XRD pattern of *a*-C film on the ITO glass as a substrate (*a*-C/ITO film). Miller indices (hkl) represents the calculated XRD pattern of ITO. The inset shows Raman spectrum of *a*-C exhibiting the D-, G-, and 2D-peaks. (**b**) Morphology images of the *a*-C/ITO film showing a dominant C element obtained by SEM–EDX. (**c**) The side view of the *a*-C/ITO, *a*-C:N/ITO, and *a*-C:B/ITO films along with illustrations of the film lining.

Figure 1a shows a representative of X-ray diffraction (XRD) patterns of *a*-C film on the ITO substrate. Miller indices of (211), (222), (400), and (440) represent peaks of ITO crystal. A characteristic of the amorphous background is observed in the films, indicated with a broad peak at 2*θ* between 15 and 35°. The inset in Fig. 1a is a Raman spectrum of *a*-C, showing the existence of G-, D-, and 2D peaks. The G-peak at 1582 cm^−1^signifies a graphitic signature of *a*-C with *sp*^2^ hybridization, while the D-peak at 1393 cm^−1^ represents disorders due to defects induced on the *sp*^2^ hybridized *a*-C sheet^31^. The broad 2D peak with a low intensity is observed at ~ 2700 cm^−1^, indicating the second order of the disorder mode^31^. The intensity of G-peak is higher than that of the D-peak with the ratio of *I*_D_/*I*_G_ ≈ 0.76. This means that a graphene-like characteristic remains in the obtained *a*-C. A cross-sectional image of each film observed using SEM along with its lining illustrations is presented in Fig. 1c. The thickness of *a*-C, *a*-C:N, and *a*-C:B films is estimated to be approximately 180, 130, and 370 nm, respectively. Microstructure of the film surface is shown in Fig. 1b, and SEM–EDX analysis confirms the existence of carbon when *a*-C deposited on the ITO substrate.

**Figure 2:**
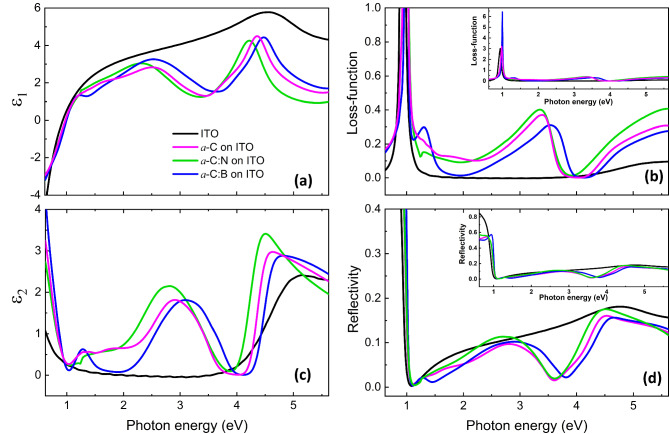
Results of the spectroscopic ellipsometry (SE). (**a**) The real part (*ε*_1_) and (**c**) imaginary part (*ε*_2_) of the complex dielectric function, (**b**) loss function, and (**d**) reflectivity of *a*-C films.

Figure 2 displays the real part (*ε*_1_) and imaginary part (*ε*_2_) of the complex dielectric function, loss function, and reflectivity of *a*-C films. The value of *ε*_1_ changes dramatically from positive to negative, indicating an insulator to metal transition crossover at the screened plasma frequency, signing a metal characteristic for the negative *ε*_1_. The *ε*_1_ reaches a minimum with a positive value at ~ 3.50 eV. A trough is observed at ~ 3.47 eV for *a*-C and *a*-C:N films and at ~ 3.72 eV for the *a*-C:B film. A metal feature is also observed in *ε*_2_ showing a Drude response at photon energy < 1 eV. All *a*-C films exhibit peaks at ~ 1.30, ~ 3.00 and ~ 4.60 eV, signifying the present of resonant excitons at these photon energies. The rise at ~ 4.60 eV is a characteristic of resonant exciton in graphene^10,23^. Another peak at ~ 3.00 eV shifts by N and B doping. It is observed at ~ 2.90, ~ 2.82, and ~ 3.10 eV for *a*-C, *a*-C:N, and *a*-C:B, respectively. This ~ 3.00 eV peak is well fitted using Fano profile (see Fig. S-6), confirming that the resonant exciton shows Fano characteristics^10,23^. The nearly absence of *ε*_2_ at ~ 3.50 to 4.00 eV shows a low plasmon loss and transparent feature in the range of mid-deep UV.

Nearly zero reflectivity is observed in *a*-C films at photon energy of ~ 1.00 and ~ 3.50 eV. The substrate also has non-reflective characteristic at ~ 1.00 eV but exhibits non-zero reflectivity at ~ 3.50 eV. When *a-*C films deposited on the substrate, the reflectivity approaches zero and shows a deep at ~ 3.50 eV, where its emergence slightly shifts by doping. The local minimum in the reflectivity at ~ 3.50 eV is a sign of the presence of correlated-plasmon^24,32^. Loss function curves indeed corroborate that correlated-plasmon appears in the films, signified by a peak at ~ 3.50 eV. The peak shows blue and red shifts as far as 140 meV by B and N doping, respectively, consistent with that of the deep in reflectivity. Another peak is also observed at ~ 1.30 eV, which implies that correlated-plasmon also presents at this photon energy. Aside from that, a conventional plasmon is confirmed to emerge at ~ 1.00 eV^33–36^, marked with a sharp peak at ~ 0.94 eV for ITO, ~ 0.98 eV for *a*-C and *a*-C:N, and ~ 1.00 eV for *a*-C:B. The presence of conventional plasmon indicates that metal characteristic of the substrate remains in films.

The SE result shows two main observations, resonant exciton and correlated plasmon, that coexist in a film consisting of semiconducting *a*-C film on metallic ITO substrate. Interestingly, the obtained film exhibits PV characteristic having significantly improved efficiency compared with existing biomass-based PVCs^37–39^. The PVC fabricated from our *a*-C film has the efficiency of 0.0708% (see the supplementary file), which is larger by three order magnitude than that previously reported of similar *a*-C films made from camphor oil (0.000048%)^38^. We should note that the cell configuration is different: the previous report is a heterojunction film with the Au/*a*-C/p-Si/Au configuration, while our film has the *a*-C:B/*a*-C/*a*-C:N/ITO configuration without any silicon in it. All *a*-C:B, *a*-C:N, and *a*-C films are made from palmyra sap. Thus, our current study presents the first homojunction film of *a*-C to show PV characteristic. This finding reveals that resonant exciton and correlated plasmon are necessary and main ingredients in generating PV characteristic in solar cell technologies. The existence of resonant excitons and correlated plasmons in *a*-Si:H film and their coupling yields new *correlated* plexcitons to enhance the power conversion efficiency of PV devices^40^.

**Figure 3:**
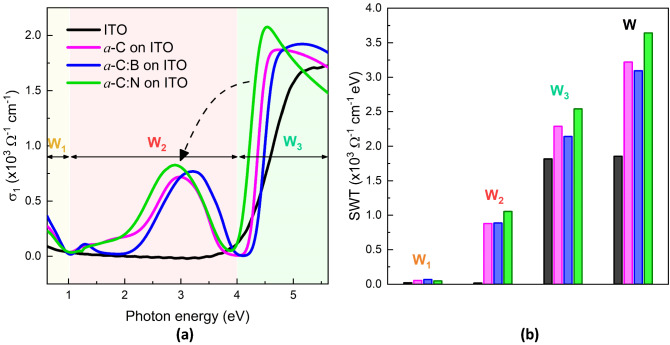
(**a**) Optical conductivity (*σ*_1_) spectra of *a*-C films on ITO substrate. (**b**) The estimated spectral-weight-transfer (SWT) over three energy ranges: 0.62–1.00 eV (W_1_), 1.00–4.00 eV (W_2_), 4.00–5.62 eV (W_3_). W = W_1_ + W_2_ + W_3_.

Optical conductivity of the films is calculated from the dielectric function, σ_1_(ω) = ε_0_ε_2_(ω)ω^24,41^. The conductivity satisfies the charge conservation, *f*-sum rule, and is related to the total electron density *n*, where ${\int }_{0}^{\infty }{\sigma }_{1}\left(\omega \right)d\omega =\frac{\pi n{e}^{2}}{2{m}_{e}}$ ^24,41^. Figure 3a shows σ_1_(ω) of the films and substrate. While the substrate has a metallic characteristic, *a*-C films show the existence of three peaks at ~ 1.30, ~ 3.00, and ~ 4.60 eV. The peak at ~ 4.60 eV is the feature of the resonant exciton present in graphene^23^, which shows that *a*-C films have graphene-*like* characteristics. Interestingly, resonant excitons also emerge at about ~ 1.30 and ~ 3.00 eV in *a*-C films. Photon energy at which the peaks indicating the appearance of resonant excitons is shifted upon doping. N-doping tends to lower the energies, while B-doping is likely to increase them.

To estimate the effective number of electrons participating in the optical transition, spectral weight transfer (SWT) is calculated within the energy range of [*E*_1_, *E*_2_], $\mathrm{W}={\int }_{{E}_{1}}^{{E}_{2}}{\sigma }_{1}\left(E\right)dE$. The SWT is a fingerprint of electronic correlations ^24,41,42^. Three energy ranges are employed, namely W_1_ (0.62–1.00 eV), W_2_ (1.00–4.00 eV), and W_3_ (4.00–5.62 eV), with the total SWT, W = W_1_ + W_2_ + W_3_. Figure 3b shows the SWT of the ITO film (substrate) and *a*-C films on the substrate. The ITO film exhibits a dominant W_3_ at *E* > 4.00 eV. The total W shows that SWT significantly increases when ITO film coated with *a*-C. It then decreases with B doping (*a*-C:B), while increases with N doping (*a*-C:N). This is related to the fact that B (1*s*^2^ 2*s*^2^ 2*p*^1^) has one electron less whilst N (1*s*^2^ 2*s*^2^ 2*p*^3^) has one more electron than C (1*s*^2^ 2*s*^2^ 2*p*^2^). The result proves that charge carries of hole and electron are introduced by B and N doping into *a*-C films, respectively.

**Figure 4:**
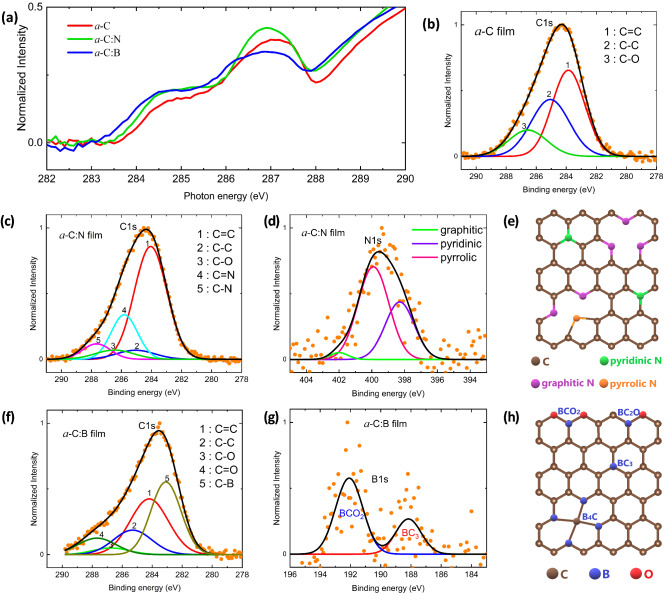
(**a**) C *K*-edge XAS spectra of the films at the photon energy range of 282–290 eV. The normalized XPS C1s spectra of (**b**) *a*-C, (**c**) *a*-C:N, and (**f**) *a*-C:B films on ITO substrate. (**d**) and (**g**) are B1s spectrum of the *a*-C:B film and N1s spectrum of the *a*-C:N film, respectively, with illustrations of C-N (**e**) and C-B (**h**) bonds at the graphenic structure.

Spectra of x-ray absorption (XAS) and photoemission spectroscopies (XPS) are presented in Fig. 4. Results of deconvoluted peaks of XPS spectra are summarized in Table S-2 (supplementary file). The XAS intensity at ~ 284 eV increases for both *a*-C:N and *a*-C:B, which means either an increase in the hybridization strength or a decrease in the number of electrons. As XPS analysis shows that C = C intensity increases in *a*-C:N while decreases in *a*-C:B, it implies that there is an enhancement in the hybridization strength of C = C in *a*-C:N and an increment in the number of electrons in *a*-C:B. At ~ 287 eV of XAS, the intensity increases for *a*-C:N while decreases for *a*-C:B. In the case of *a*-C:N, this relates with the presence of C=N and C–N bonds. Thus, intensity drops in *a*-C:B is due to the absence of these bonds. Furthermore, XAS spectra of *a*-C:B shifts to low energy at ~ 283 eV, which confirms the existence of the C-B bond. The XAS intensity also increases at ~ 285 eV for both *a*-C:N and *a*-C:B films. As the relative intensity of C–C in XPS spectra decreases in the films, it means that the number of electrons at C–C also decreases.

**Figure 5:**
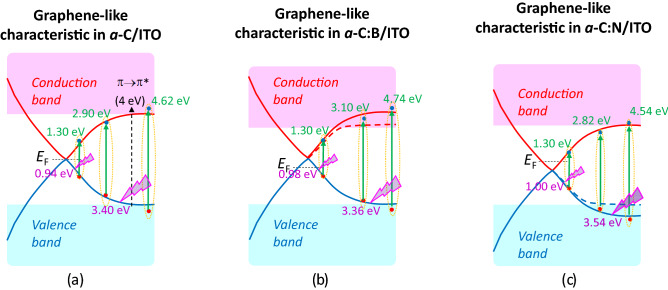
Schematics band diagram of graphene-*like* characteristic in (**a**) *a*-C/ITO film, (**b**) *a*-C:B/ITO film, and (**c**) *a*-C:N/ITO film. The resonant excitonic effects are present in the films.

Referring to the presented data, we propose band structure models of graphene-*like* characteristic in the *a*-C/ITO, *a*-C:B/ITO, and *a*-C:N/ITO, as shown in Fig. 5. The *a*-C film has graphene-*like* characteristic, having a Dirac cone-like feature at Fermi level. While a band transition of π–π* is observed at ~ 4 eV, the resonant excitonic effects present at ~ 1.00, ~ 3.00, and ~ 4.60 eV. As ITO bands are close to *a*-C bands, interactions between *p*-band of graphene-*like* film and *sp*-band hybridization of ITO could be realized. In the *a*-C:B/ITO, hole doping in the *a*-C film creates a new state below the conduction band with energy gap of 2–3 eV, while electron doping creates one above the valence band. Moreover, effects of resonant excitonic are also observed in the *a*-C:B and *a*-C:N films, where its energy is affected by doping.

## Conclusion

Roles of exciton and plasmon is crucial to understand a fundamental mechanism that generates power conversion efficiency of PV devices. In the current study, we observe the coexistence of resonant excitons and correlated plasmons in amorphous carbon films. Amorphous carbon (*a*-C) is prepared from palmyra sugar, and *a*-C films are fabricated using a nano-spraying method. This procedure offers simple, environmentally friendly, and reproducible technique. Structure analysis shows that a graphene-*like* characteristic remains in the obtained *a*-C film. The loss function, reflectivity, and real part (*ε*_1_) of the complex dielectric functions signify the present of correlated plasmons at ~ 1.30 and ~ 3.50 eV, while the imaginary part (*ε*_2_) of the complex dielectric function and the optical conductivity (*σ*_1_) indicate the existence of resonant exciton at ~1.30, ~3.00 and ~ 4.60 eV. Moreover, the introduction of hole and electron as charge carriers in *a*-C film is successfully performed by B and N doping, respectively, which is confirmed by spectral weight transfer (SWT) estimation and XAS and XPS analyses. The doping shifts photon energies of the emergence of correlated plasmons and resonant excitons. The coupling between resonant excitons and correlated plasmons yields a *correlated* plexciton that is essential to enhance the power conversion efficiency of PVCs. Lastly, by referring to all presented data, band structure models of graphenic-*like* characteristic in *a*-C/ITO, *a*-C:B/ITO, and *a*-C:N/ITO are proposed.

## Supplementary Information

Supplementary Information.

## Data Availability

All data generated or analysed during this study are included in this published article [and its supplementary information files].
