# Supplementary material for: Unrevealing tunable resonant excitons and correlated plasmons and their coupling in new amorphous carbon-like for highly efficient photovoltaic devices
Source: Sci Rep. 2023 May 4;13:7262. doi: 10.1038/s41598-023-31552-5 (PMC10160088; doi:10.1038/s41598-023-31552-5)
Supplement: Supplementary file 1 — Supplementary Information. [file 41598_2023_31552_MOESM1_ESM.docx]

Supplementary Information

**Unrevealing tunable resonant excitons and correlated plasmons and their coupling in new amorphous carbon‑*like* for highly efficient photovoltaic devices**

D. Darminto^1,*^, Retno Asih^1^, Budhi Priyanto^1,2^, Malik A. Baqiya^1^, Irma S. Ardiani^1^,

Khoirotun Nadiyah^1^, Anna Z. Laila^1^, Soni Prayogi^1^, Sarayut Tunmee^3^, Hideki Nakajima^3^,

Angga D. Fauzi^4,5^, Muhammad A. Naradipa^4,5^, Caozheng Diao^5^ & Andrivo Rusydi^4,5,*^

^1^Department of Physics, Institut Teknologi Sepuluh Nopember, Surabaya 60111, Indonesia. ^2^Department of Electrical Engineering, Muhammadiyah University, Malang 65145, Indonesia. 3Synchrotron Light Research Institute, 111 University Avenue, Muang District, Nakhon Ratchasima 30000, Thailand.

^4^ Advanced Research *Initiative* for Correlated-Electron Systems (AR*i*CES), Department of Physics, National University of Singapore, Singapore 117542, Singapore.

^5^Singapore Synchrotron Light Source, National University of Singapore, 5 Research Link, Singapore 117603, Singapore.

*email: darminto@physics.its.ac.id; andrivo.rusydi@nus.edu.sg

Amorphous carbon (*a*-C) is synthesized from palmyra sap, a clear liquid that comes out of the flowers of the palmyra tree. Palmyra sap can be consummed as it is or processed to produce palmyra sugar. Figure S-1 presents images of palmyra tree and palmyra sugar. The *a*-C films are then prepared by depositing *a*-C liquid on the substrate (ITO on glass). H_3_BO_3_ (Merck, 99.5%) and NH_4_OH 1 M (Merck, 25%) are used to introduce B and N doping into *a*-C, respectively. Detailed procedures are desrcibed in the manuscript. XRD patterns of *a*-C, *a*-C:B, and *a*-C:N on the substrate are also shown in Fig. S-1, showing diffraction peaks of ITO with a broad peak at ~25° as a background. A broad peak might confirm an amorphous feature of *a*-C.


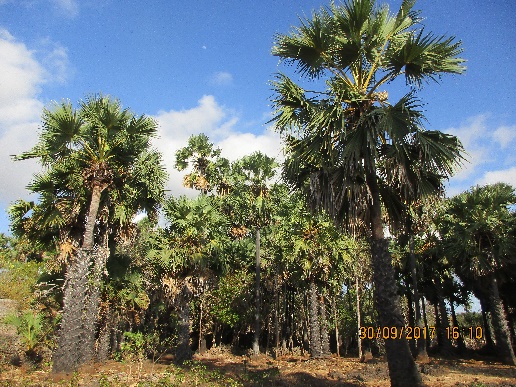

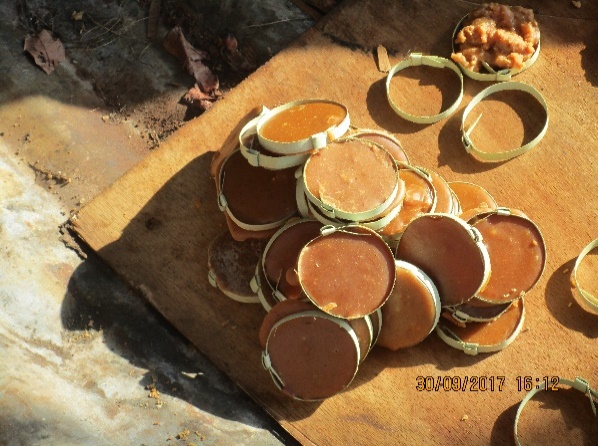


**Figure S-1**: Supplementary Figure. (Left) Palmyra tree and palmyra sugar made from palmyra sap that is used to prepare amorphous carbon (*a*-C). (Right) XRD patterns of *a*-C, *a*-C:B, and *a*-C:N films on ITO substrate. The (hkl) represents diffraction peaks of ITO.

Figure S-2 shows the Ψ and 𝚫 curves of the ITO film on glass that is used as the substrate to prepare *a*-C films. The Ψ and 𝚫 parameters are gathered at incident angles of 50°, 60°, and 70° and are collected at room temperature within the photon energy range of 0.6 - 6.2 eV.

**
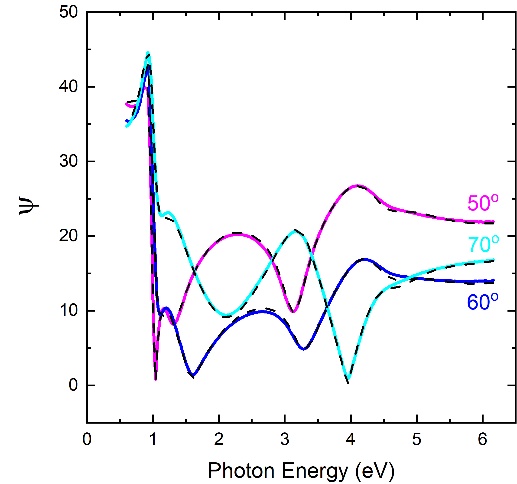
**
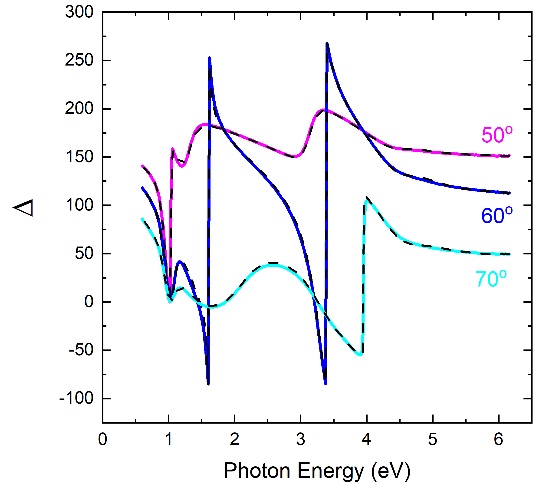


**Figure S-2**: Supplementary Figure. Experimental (solid curves) and fitted (dotted curves) data of Ψ and 𝚫 of ITO on glass as the substrate.


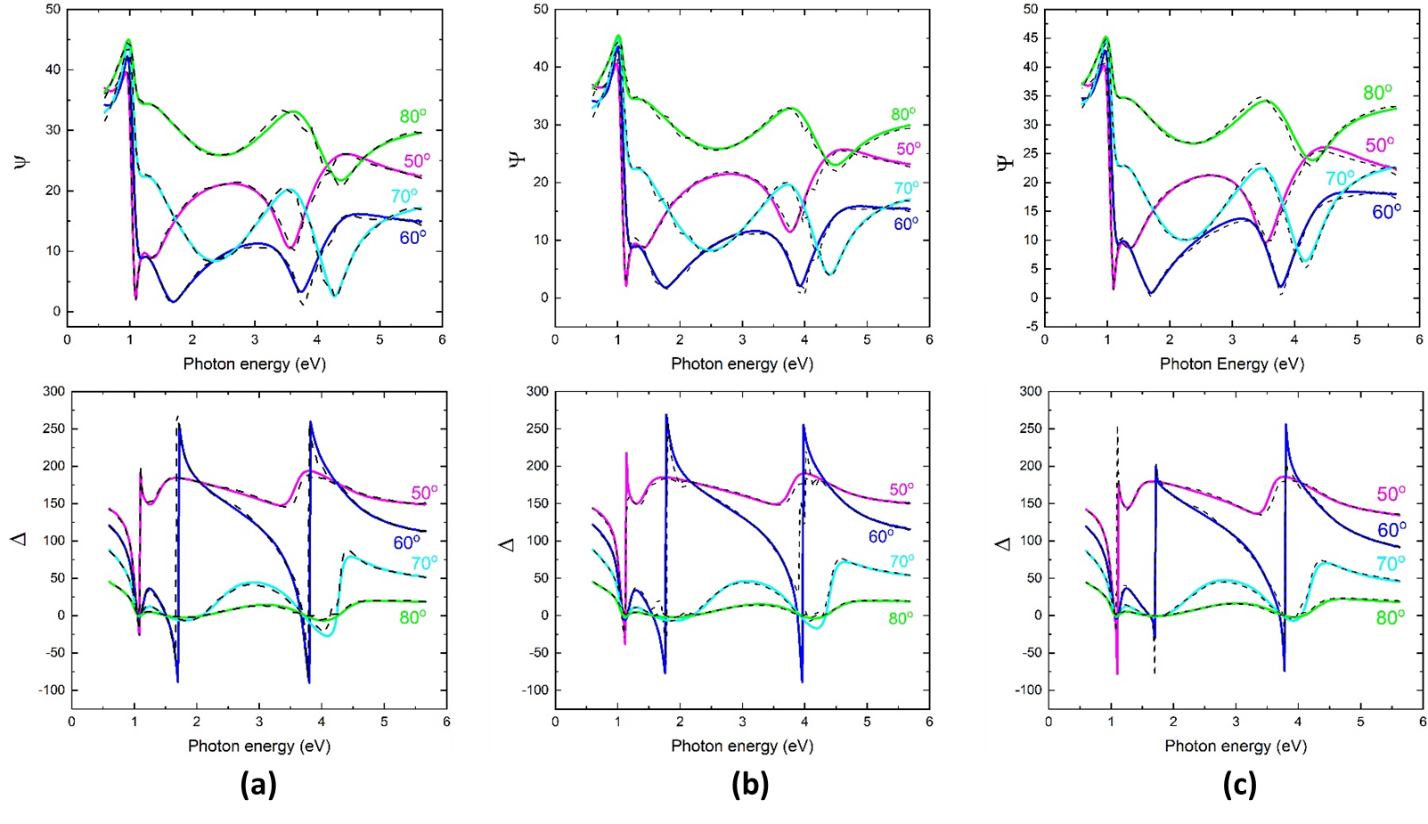
**Figure S-3**: Supplementary Figure. Experimental (solid curves) and fitted (dotted curves) data of Ψ and 𝚫 of *a*-C/ITO, *a*-C:B/ITO, and *a*-C:N/ITO films.

The SE measurements are performed using a Woollam V-vase ellipsometer. Details of the apparatus can be seen elsewhere^1^. The glass is well fitted using a Cauchy model, and the ITO film is finely fitted using a Gen-Osc model. The resulted fittings are illustrated as the dotted curves in Fig. S-2. The thickness of ITO film is found to be 107.1 ± 0.1 nm with 7.7 ± 0.1 nm roughness and (1.94 ± 0.1)% of inhomogeneity. The obtained parameters of the substrate are then fixed to examine dielectric properties of *a*-C films. The Ψ and 𝚫 data of *a*-C films: *a*-C/ITO, *a*-C:B/ITO, and *a*-C:N/ITO, are presented in Fig. S-3. The data is gathered at 50°, 60°, 70°, and 80° of incident angles at photon energy of 0.62 to 5.62 eV. The best fitting results are achieved by employing a Gen-Osc model with six PSemi-Tri functions and are represented as dotted curves in Fig. S-3.

From Ψ and 𝚫 parameters, one can obtain parameters related with dielectric properties as follows^1^:

- Complex dielectric function, $\varepsilon\left( \omega\right)=\varepsilon_{1}\left( \omega\right)+i\varepsilon_{2}\left( \omega\right)$; $\omega$ is the angular frequency of the incident photon.
- Complex refractive index, $\tilde{n}=n\left( \omega\right)+i k\left( \omega\right)$ , in which

$n$ is the refractive index, $n(\omega)=\sqrt{\frac{1}{2}\left[ \left( \varepsilon_{1}^{2}\left( \omega\right)+\varepsilon_{2}^{2}\left( \omega\right) \right)^{1/2}+\varepsilon_{1}\left( \omega\right) \right]}$and

$k$ is the extinction coefficient, $k(\omega)=\sqrt{\frac{1}{2}\left[ \left( \varepsilon_{1}^{2}\left( \omega\right)+\varepsilon_{2}^{2}\left( \omega\right) \right)^{1/2}-\varepsilon_{1}\left( \omega\right) \right]}$

- Normal incident reflectivity, $R\left( \omega\right)=\frac{{[n\left( \omega\right)-1]}^{2}+k^{2}(\omega)}{{[n\left( \omega\right)+1]}^{2}+k^{2}(\omega)}$
- Loss function, $-\mathrm{Im}\left[ \varepsilon^{-1}\left( \omega\right) \right]=\frac{\varepsilon_{2}(\omega)}{\left[ {\varepsilon_{1}}^{2}\left( \omega\right)+{\varepsilon_{2}}^{2}(\omega) \right]}$
- Optical conductivity, $\sigma_{1}\left( \omega\right)=\varepsilon_{0}\varepsilon_{2}(\omega)\omega$; $\varepsilon_{0}$ is the free-space permittivity.

The obtained data of the real part (*ε*_1_) and imaginary part (*ε*_2_) of the complex dielectric function, loss function, and reflectivity of *a*-C/ITO, *a*-C:B/ITO, and *a*-C:N/ITO are shown in Fig. 2. Analyses of the first and second derivatives of the complex dielectric function are performed to further support the discussion of the existence of resonant excitons and correlated plasmons described in the manuscript.


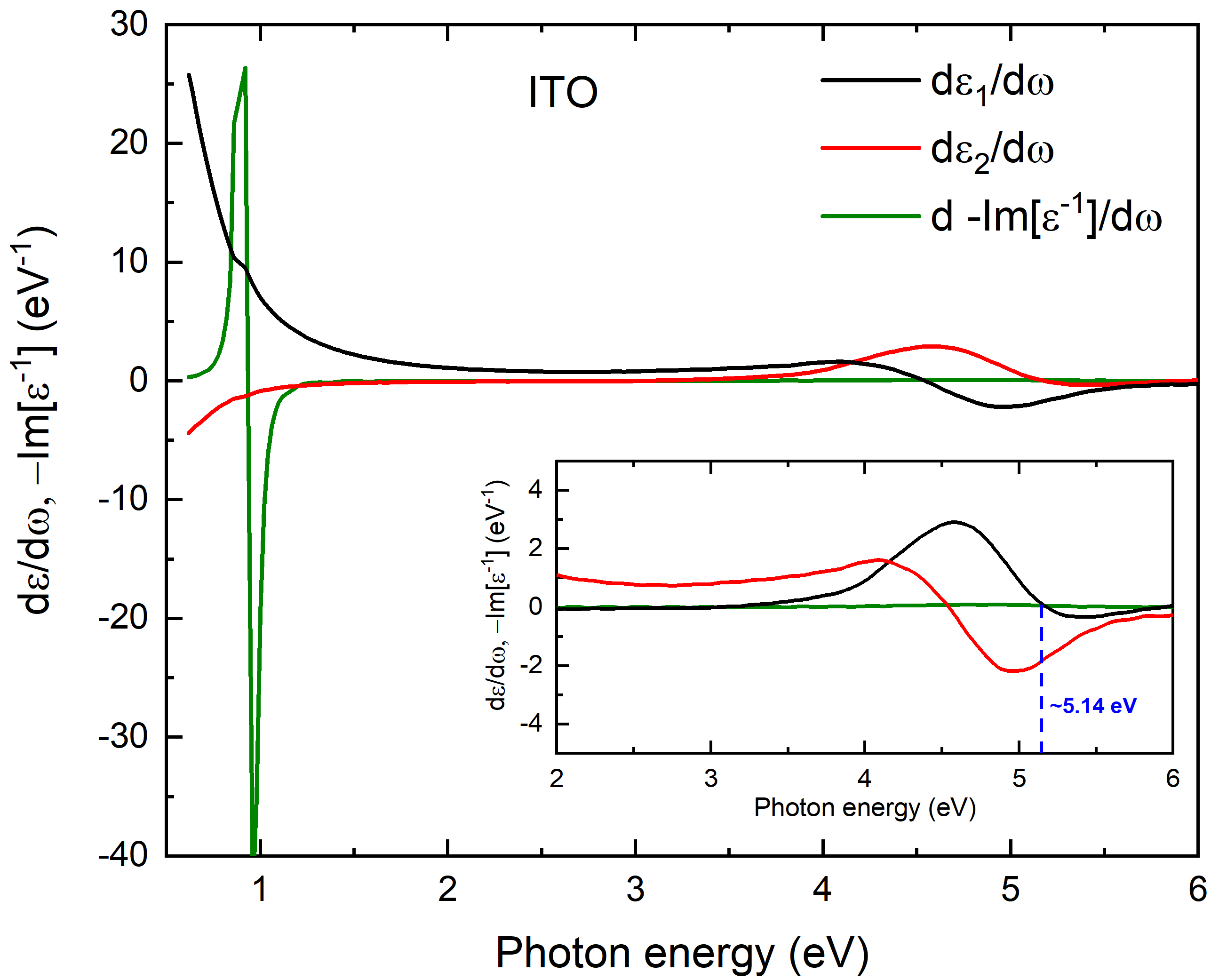

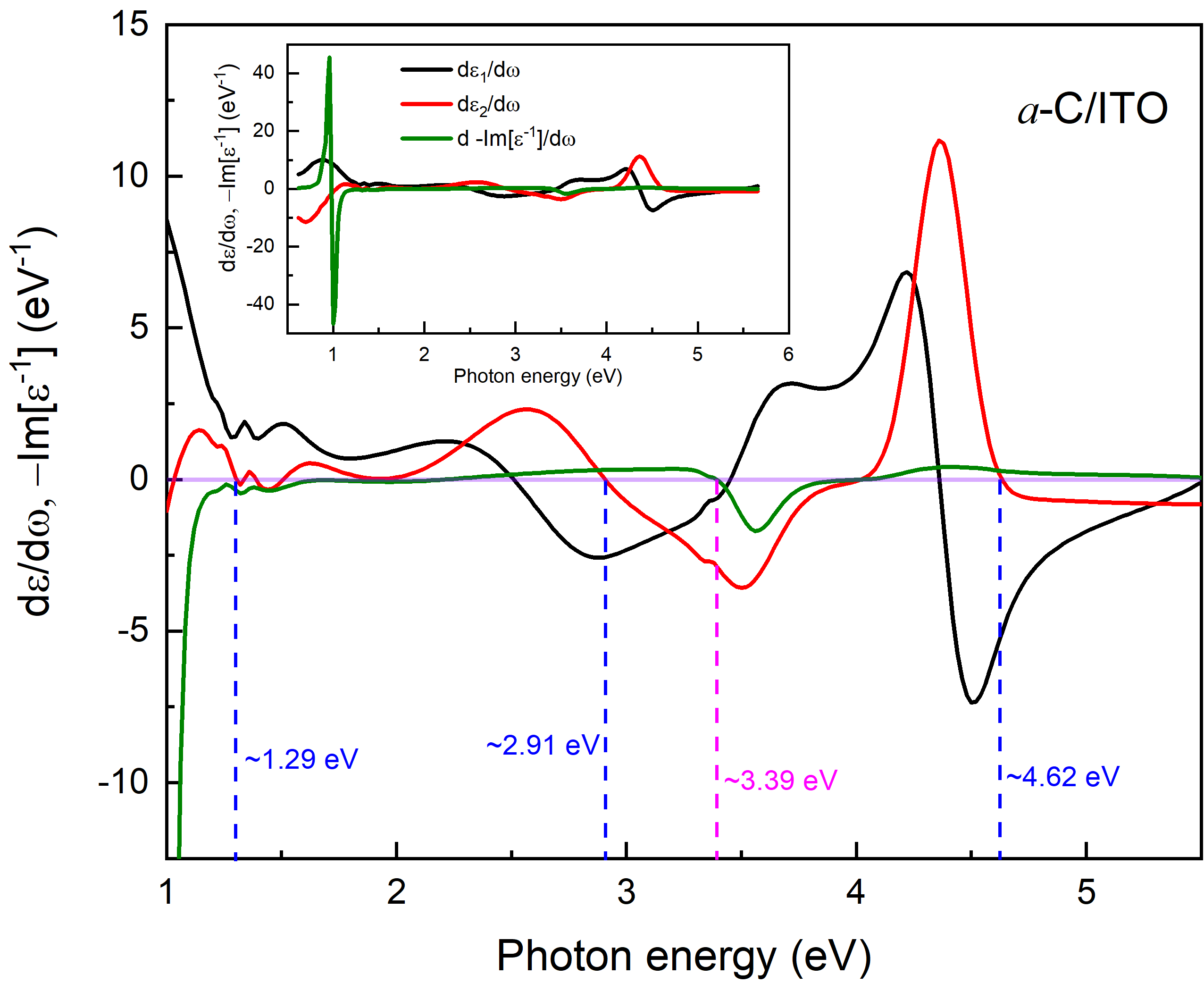


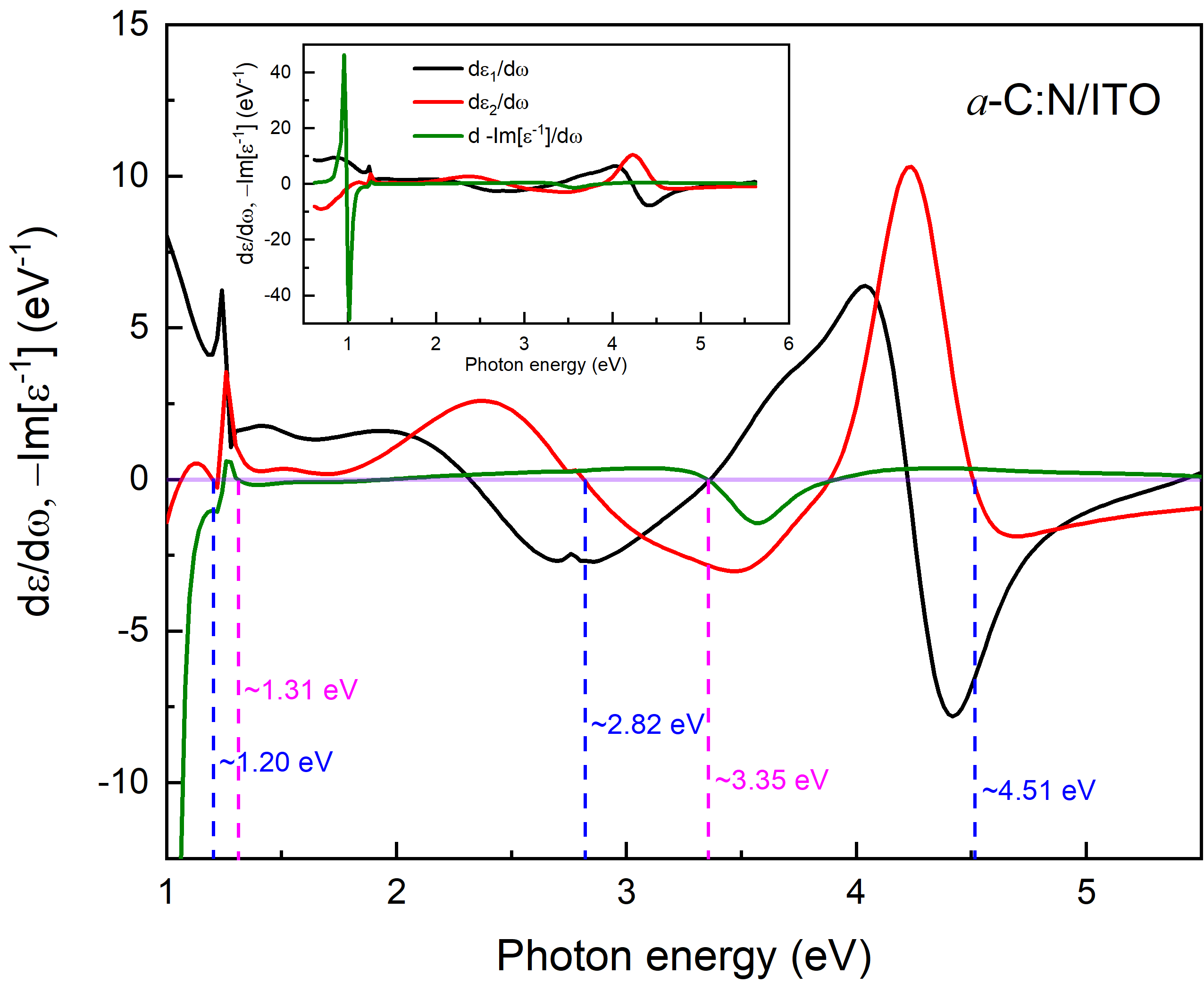

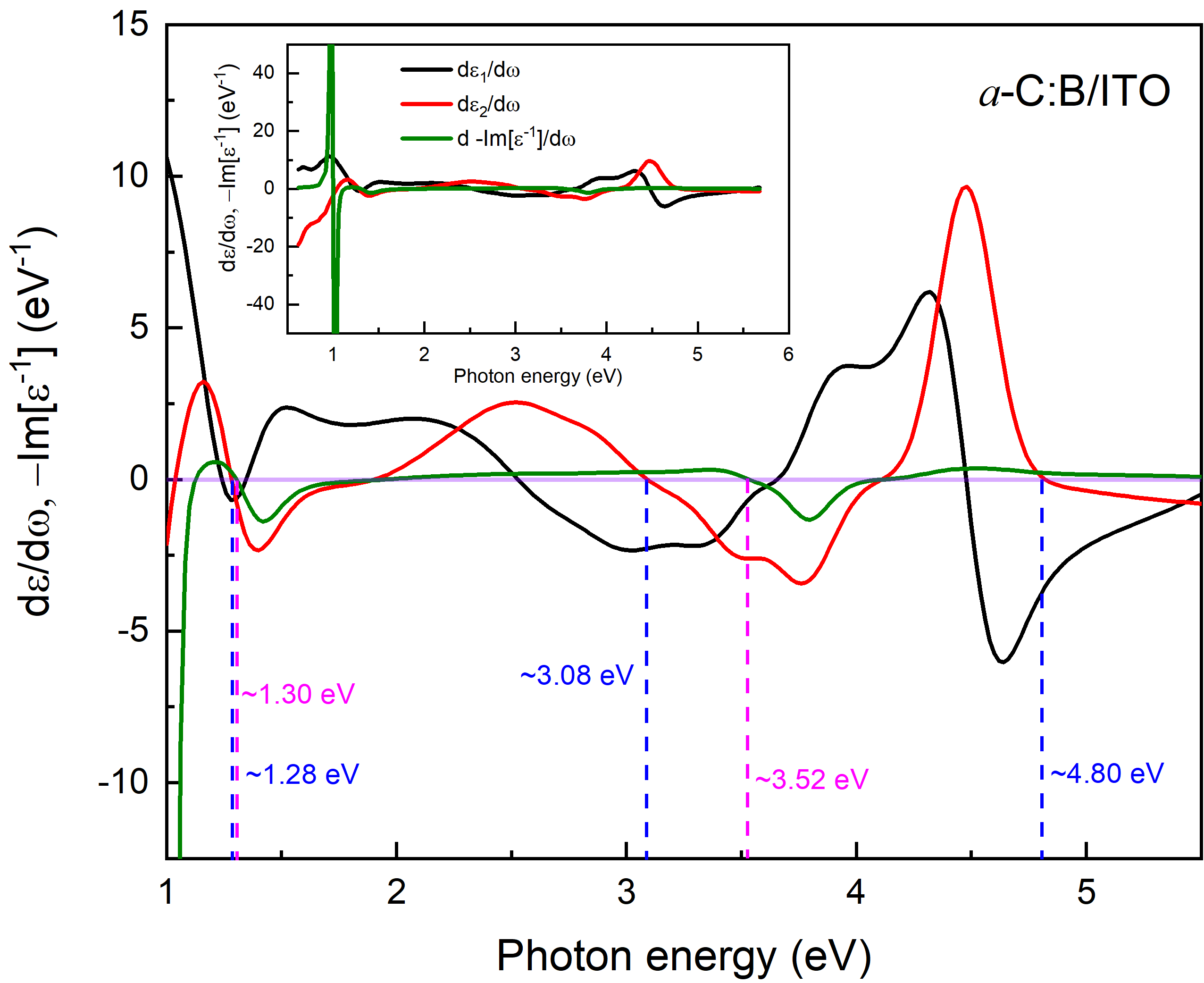


**Figure S-4**: Supplementary Figure. The first derivative of <*ε*_1_>, <*ε*_2_>, and loss function (-Im[*ε*^-1^]) of ITO, *a*-C/ITO, *a*-C:N/ITO, and *a*-C:B/ITO films. Insets show full-scale graphs for clarity.

Figure S-4 presents the first derivatives of <*ε*_1_>, <*ε*_2_>, and loss function (-Im[*ε*^-1^]) of ITO, *a*-C/ITO, *a*-C:N/ITO, and *a*-C:B/ITO films. A crossing zero in the d*ε*_2_/d*ω*, which is related with resonant excitons, is observed in all *a*-C films at ~1.3, ~3.0, and ~4.6 eV. The exact photon energy shifts by N and B doping. It is obvious that the crossing zero feature at these photon energies is absent in the ITO substrate. Instead, a crossing zero of the ITO film occurs at ~5.1 eV. The d-Im[*ε*^-1^]/d*ω* of the *a*-C/ITO film shows crossing-zero attributes at ~3.4 eV that also shift by doping. Another crossing-zero characteristic is observed at ~1.3 eV. One should also note that this feature is absent in the ITO substrate. The ITO film exhibits a typical symmetric line-shape of the d-Im[*ε*^-1^]/d*ω* at ~1.00 eV that remains in all *a*-C films. This confirms that a conventional plasmon appears at ~1.00 eV in all films. Hence, the features at ~1.3 and ~3.4 eV are related with so-called correlated plasmons.


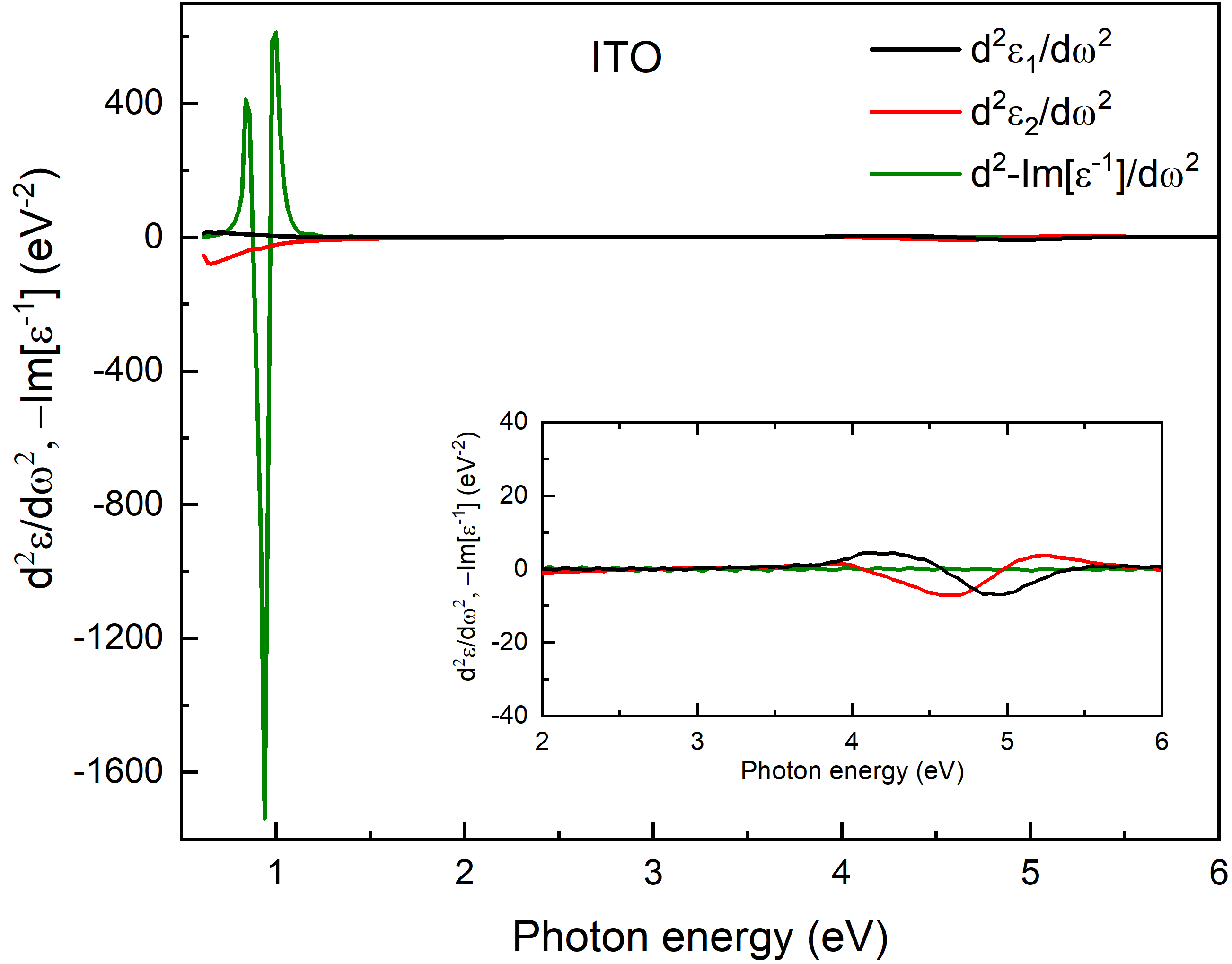

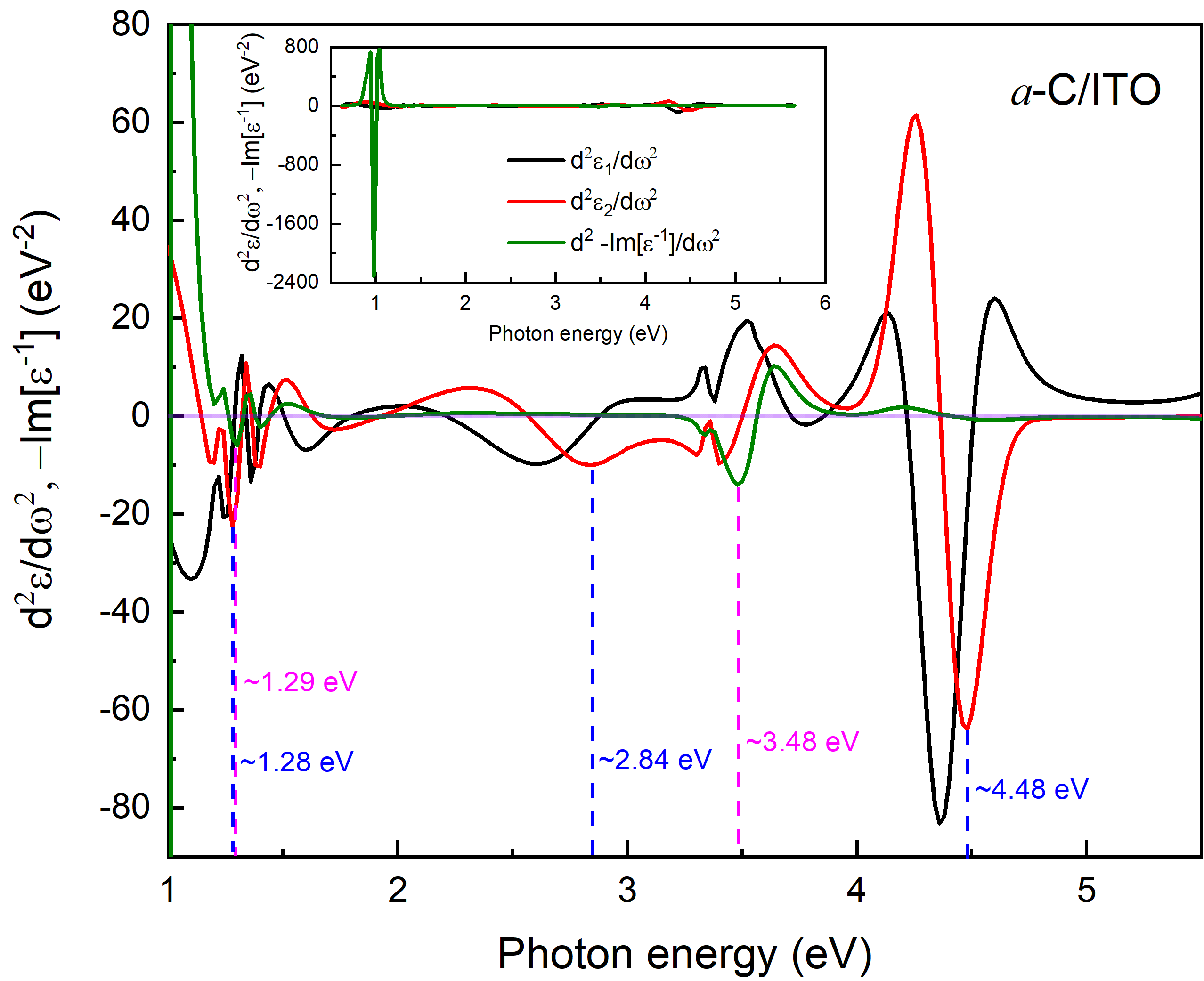


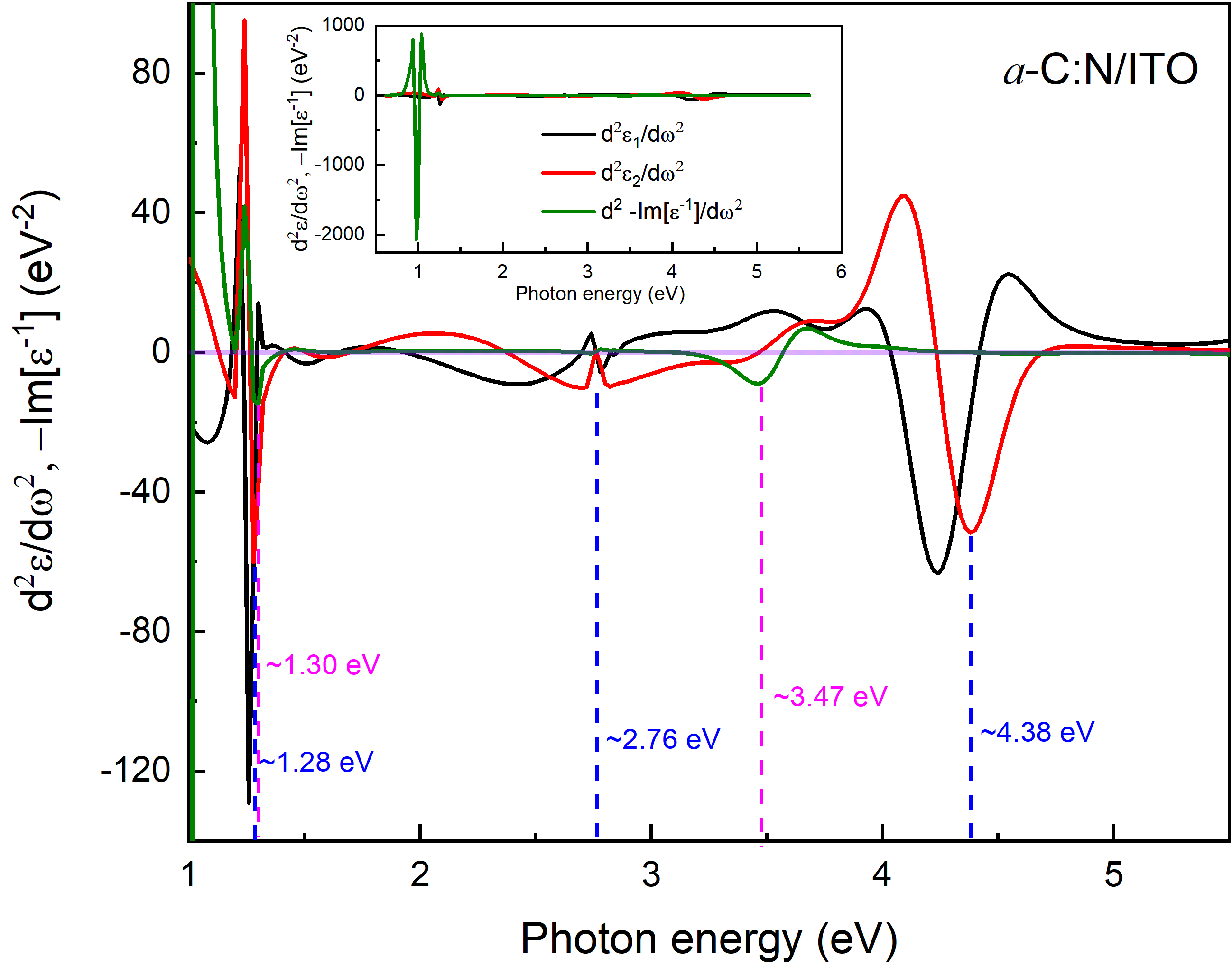

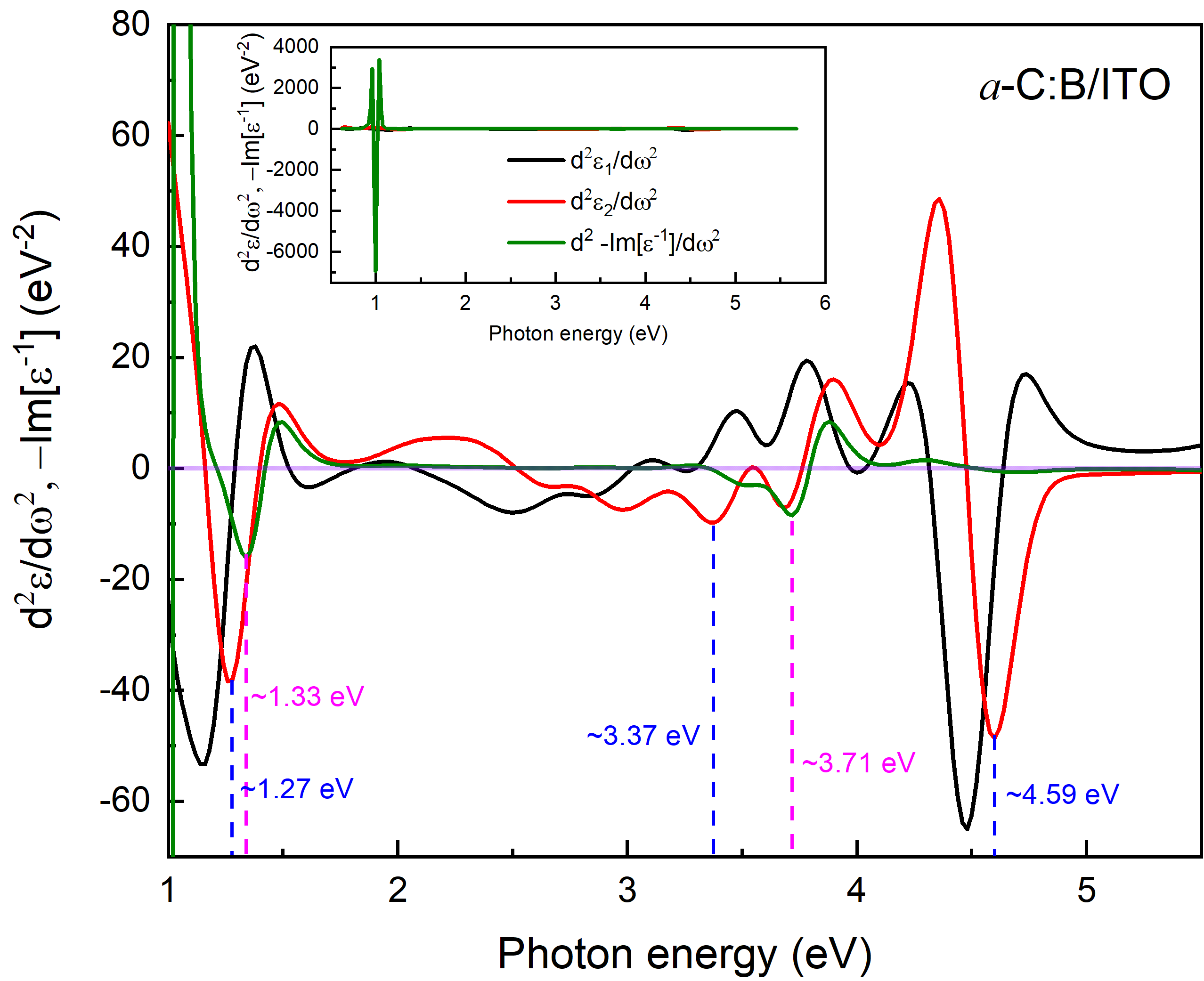


**Figure S-5**: Supplementary Figure. The second derivative of <*ε*_1_>, <*ε*_2_>, and loss function (-Im[*ε*^-1^]) of ITO, *a*-C/ITO, *a*-C:N/ITO, and *a*-C:B/ITO films. Insets show full-scale graphs for clarity.

The second derivative of <*ε*_1_>, <*ε*_2_>, and loss function (-Im[*ε*^-1^]) of ITO, *a*-C/ITO, *a*-C:N/ITO, and *a*-C:B/ITO films with respect to photon energy are displayed in Fig. S-5. Peaks in the second derivatives of <*ε*_2_> and loss function appear at photon energy that is nearly consistent with that at where the crossing-zero feature takes place. This further confirms the present of resonant excitons and correlated plasmons in the *a*-C films.

The resonant exciton at ~3.0 eV can be fit with the Fano profile, as shown in Fig. S-6. Fano line shape is described as $I\propto\frac{{(q+\varepsilon)}^{2}}{1+\varepsilon^{2}}$ with $\varepsilon=\frac{2(\omega-\omega_{0})}{\Gamma}$, hence $I\propto\frac{\left( q\Gamma+2(\omega-\omega_{0}) \right)^{2}}{\Gamma^{2}+{(\omega-\omega_{0})}^{2}}$. The modified fitting function used is described in Eq. S1.

$y\propto\frac{A*\left( q*g+\left( x-\omega_{0} \right) \right)^{2}}{g^{2}+\left( x-\omega_{0} \right)^{2}}+BG$ (S1)

*A* and *BG* are a constant and background, respectively. *g* (or Γ) describes the line width of the resonant energy, and *q* is the Fano parameter which measures the ratio of resonant scattering to the direct (background) scattering amplitude. The values of each parameter determined from the fitting is summarized in Table 1. One can see that the peak at ~3.0 eV can be well fit with the Fano profile, as indicated by the value of the R-square and reduced chi-square. This result implies that the resonant exciton shows a Fano characteristic.


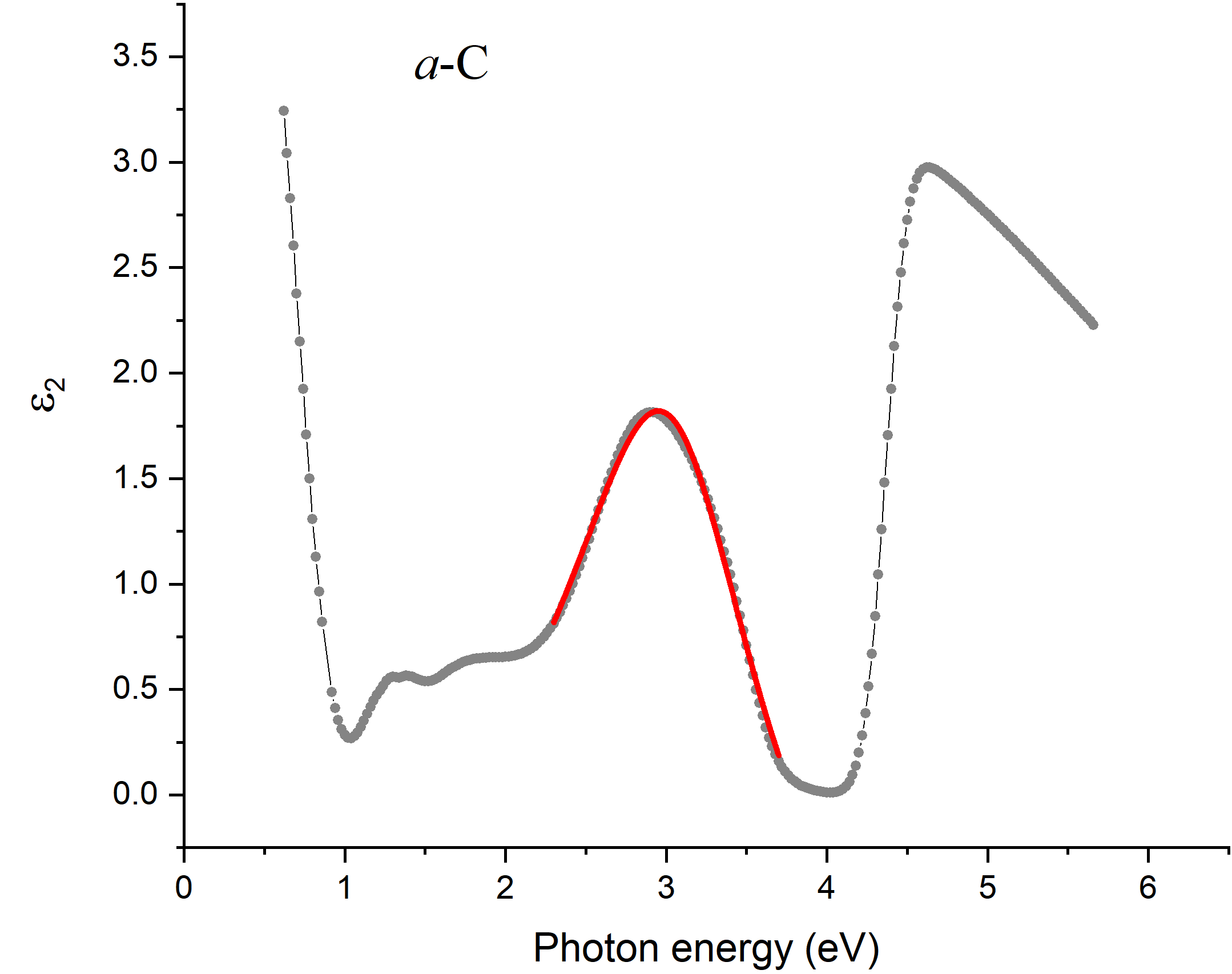


**Figure S-6**: Supplementary Figure. The <*ε*_2_> curve of *a*-C. Red line is the fit to the Fano profile at ~3.0 eV.

**Table 1**: Supplementary Table. Parameters values determined from fit of the resonant exciton (described as *ε*_2_) at 3.5 eV.

| ***A*** | 0.08018 ± 0.00885 |
| --- | --- |
| ***q*** | 5.94521 ± 0.37073 |
| ***g*** | -0.76776 ± 0.02283 |
| ***ω*_0_** | 3.08024 ± 0.01027 |
| ***BG*** | -1.09369 ± 0.09331 |
| **Reduced Chi-Sqr** | 0.00125 |
| **R-Square (COD)** | 0.99593 |
| **Adj. R-Square** | 0.99572 |

**Table 2**: Supplementary Table. Summary of XPS data obtained from deconvolution methods regarding the binding energy values, percentage of each component, and their respective assignments

| **Films** | **Spectra** | **Assignments** | **Binding energy (eV)** | **at%** |
| --- | --- | --- | --- | --- |
| *a*-C | C1s | C=C (sp^2^)  C-C (sp^3^)  C-O | 283.85  285.07  286.56 | 46.9  36.3  16.8 |
| *a*-C:N | C1s | C=C (sp^2^)  C-C (sp^3^)  C-O  C=N (sp^2^)  C-N (sp^3^) | 284.06  285.06  286.47  285.81  287.65 | 61.2  5.8  6.0  19.7  7.3 |
|  | N1s | “pyrrolic” N  “pyridinic” N  “graphitic” N | 399.90  398.30  402.00 | 36.0  61.4  2.6 |
| *a*-C:B | C1s | C=C (sp^2^)  C-C (sp^3^)  C-O  C=O  B-C | 284.19  285.31  286.76  287.68  283.08 | 34.8  15.3  4.0  10.3  35.6 |
|  | B1s | BC_3_  BCO_2_ | 188.17  192.08 | 30.3  69.7 |

Results of deconvoluted peaks of XPS spectra are summarized in Table 2, showing that the relative percentage (at%) of C=C increases in *a*-C:N while decreases in *a*-C:B. The at% of C=C (*sp*^2^) is higher than that of C-C (*sp*^3^), and oxygen functional groups (C-O) remain in all films. Moreover, the C=N and C-N bonds are observed in *a*-C:N, and the B-C bond is seen in *a*-C:B, indicating a successful N and B doping in *a*-C.

The I-V measurement is then performed to estimate the power conversion of the fabricated PV cell. The result shows that the PV cell has the efficiency of 0.0708%, which is larger than that previously reported of similar *a*-C films made from camphor oil (0.000048%)^2^. We should note that the cell configuration is different: the previous report is a heterojunction film with the Au/*a*-C/p-Si/Au configuration, while our film has the *a*-C:B/*a*-C/*a*-C:N/ITO configuration without any silicon in it. All *a*-C:B, *a-*C:N, and *a*-C films are made from palmyra sap. Our current study presents the first homojunction film of *a*-C to show PV characteristic. Figure S-7 shows the I-V curve of the p-i-n junction measured at AM 1.5 illumination. The cell configuration is illustrated in the inset.


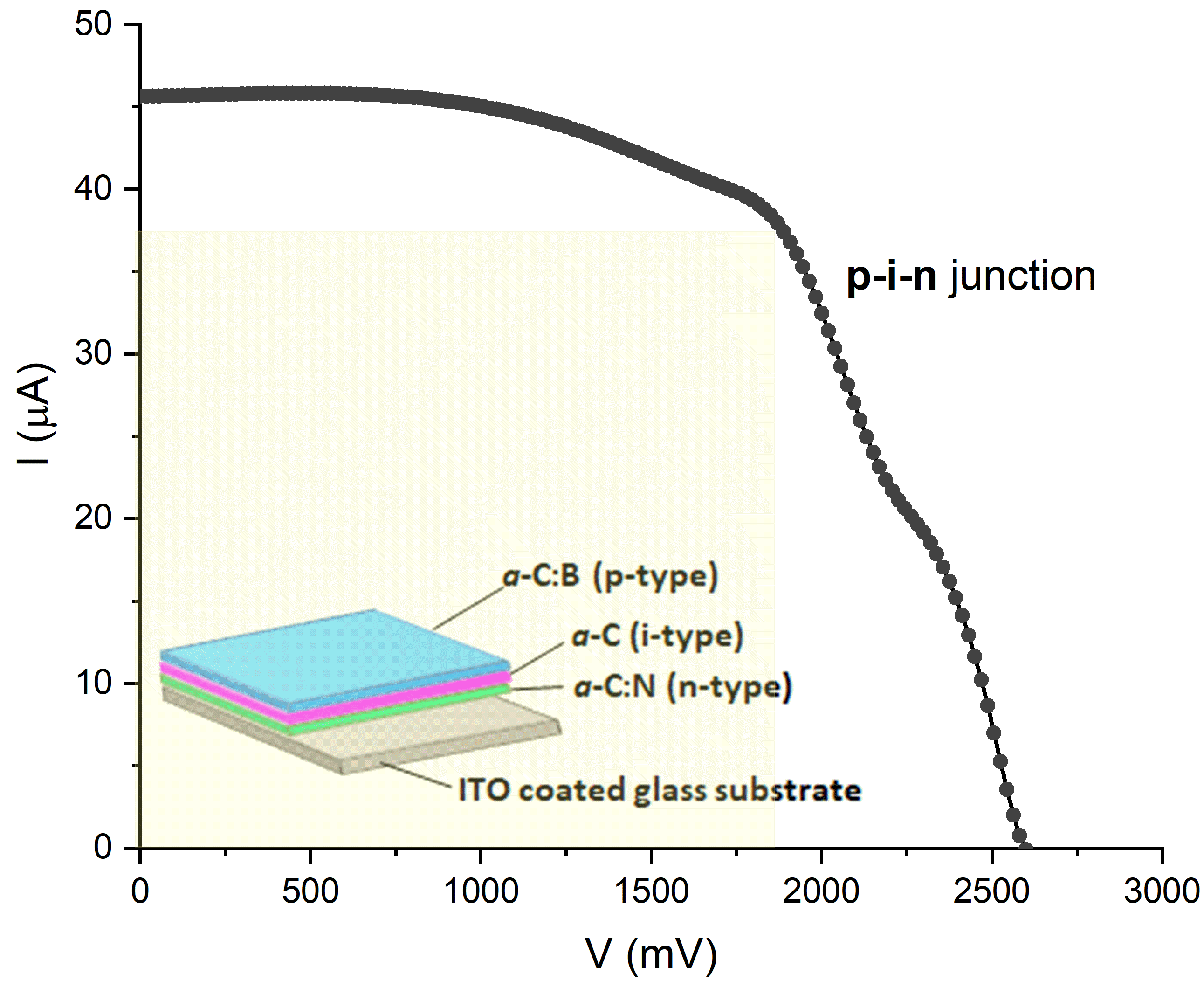


**Figure S-7**: Supplementary Figure. The I-V curve of the p-i-n junction measured at AM 1.5 illumination. Inset shows an illustration of the cell configuration.

Analysis of the I-V curves yields the results summarized in Table 3. The efficiency of approximately 0.0708% is estimated in the p-i-n junction. This value is significantly larger than that found in other biomass-derived *a*-C films. Comparison of the efficiency of the current study and the previous reports is presented in Table 4.

**Table 3**: Supplementary Table. PV characteristics of the p-i-n homojunction fabricated from amorphous carbon.

| PV characteristics | p-i-n junction |
| --- | --- |
| *I*_SC_ (μA)  *V*_OC_ (mV)  *I*_Max_ (μA)  *V*_max_ ( mV)  Fill factor (%)  Output power (mW/cm^2^)  Efficiency (%) | 45.697  2600  37.305  1900  59.656  70.88×10^-3^  0.0708 |

**Table 4**: Supplementary Table. Comparison of the current study with previous reports.

|  | Cell configurations | Deposition method | Efficiency [%] |
| --- | --- | --- | --- |
| A.N. Fadzilah, *et. al*^2^ | Au/n-C:N/p-Si/Au | Aerosol assisted CVD (AACVD) | 0.0002-0.0010* |
|  | Au/*a*-C/p-Si/Au |  | 0.000048* |
| A.N. Fadzilah, *et. al*^3^ | Au/p-C/n-Si/Au | AACVD | 0.008* |
| R.U.A. Khan, *et. al*^4^ | *a-C*:H/(i)*a-Si*:H/n-*a-Si*:H(n) | rf-PECVD | 3.84 |
| Tetsuo Soga, *et. al*^5^ | Al/C_60_/*a-C/*ITO (450°C) | Exposing N_2_ radical to sublimated C60 in the ultrahigh vacuum chamber | 0.021 |
|  | Al/C_60_/*a-C/*ITO (500°C) |  | 0.0085 |
| Tong X. Cui, *et. al*^6^ | *a*-C/graphene hybrid/Ni (400°C) | CVD | 0.003 |
|  | *a*-C/graphene hybrid/Ni (400°C-HNO_3_) |  | 0.004 |
| S. Adhikari, *et. al*^7^ | p-C/n-Si | Microwave surface wave plasma CVD | 0.16 |
|  | p-C/i-C/n-Si |  | 2.349 |
| Current study | *a-C*:B/*a-C*/*a-C*:N/ITO | Nano-spraying | 0.0708* |

*It employs biomass/bioproduct to synthesis *a*-C.

**References:**

1. Chaudhuri, A. *et al.* Quasilocal plasmons in the insulator-metal transition in the Mott-type perovskites e u0.3 B a0.7 T i1-x N bx O3. *Phys. Rev. B* **98**, 165303 (2018).

2. A.N. Fadzilah, K. Dayana and M. Rusop, Carbon based solar cell from amorphous carbon with nitrogen incorporation. *Advanced Materials Research*, vol.576, 785-789 (2012).

3. A.N. Fadzilah, K. Dayana and M. Rusop, Amorphous carbon deposited a novel aerosol assisted chemical vapor deposition for photovoltaic solar cell, *Japanese Journal of Applied Physics*, vol. 51, 06FD05 (2015).

4. R.U.A. Khan, S.R.P. Silva, R.A.C.M.M. Van Swaaij, Polimeric amorphous carbon as p-type window within amorphous silicon solar cell, *Applied Physics Letter*, vol. 82, no. 22 (2003).

5. Tetsuo Soga, Tomoya Nakagaki, Shinya Kato and Naoki Kishi, Effect of sublimation of temperature on the photovoltaic properties of amorphous carbon thin film from fullerene, *Journal of Solar Energy Research Update*, vol. 5, 8-13 (2018).

6. Tongxiang Cui, Ruitao Lv, Zheng-Hong Huang, Hongwei Zhu, Yi Jia, Shuxiao Chen, Kunlin Wang, Dehai Wu and Feiyu Kang, Low-temperature synthesis of multilayer graphene/amorphous carbon hybrid film and their potential application in solar cells, *Nanoscale Research Letters*, vol. 7, 453 (2012).

7. Sudip Adhikari, M.S. Kayastha, D.C. Gimeri, H.R. Aryal, T. Takeuchi, K. Murakami, Y. Kawashiwo, H. Uchida, K. Wakita, M. Umeno, Improved photovoltaic properties of heterojunction carbon based solar cells, *Journal of Surface Material and Advanced Technology*, vol. 3, 178-183 (2013).
